# Capturing farm diversity with hypothesis-based typologies: An innovative methodological framework for farming system typology development

**DOI:** 10.1371/journal.pone.0194757

**Published:** 2018-05-15

**Authors:** Stéphanie Alvarez, Carl J. Timler, Mirja Michalscheck, Wim Paas, Katrien Descheemaeker, Pablo Tittonell, Jens A. Andersson, Jeroen C. J. Groot

**Affiliations:** 1 Farming Systems Ecology, Wageningen University & Research, Wageningen, The Netherlands; 2 Plant Production Systems, Wageningen University & Research, Wageningen, The Netherlands; 3 CIMMYT-Southern Africa, Harare, Zimbabwe; Public Library of Science, UNITED KINGDOM

## Abstract

Creating typologies is a way to summarize the large heterogeneity of smallholder farming systems into a few farm types. Various methods exist, commonly using statistical analysis, to create these typologies. We demonstrate that the methodological decisions on data collection, variable selection, data-reduction and clustering techniques can bear a large impact on the typology results. We illustrate the effects of analysing the diversity from different angles, using different typology objectives and different hypotheses, on typology creation by using an example from Zambia’s Eastern Province. Five separate typologies were created with principal component analysis (PCA) and hierarchical clustering analysis (HCA), based on three different expert-informed hypotheses. The greatest overlap between typologies was observed for the larger, wealthier farm types but for the remainder of the farms there were no clear overlaps between typologies. Based on these results, we argue that the typology development should be guided by a hypothesis on the local agriculture features and the drivers and mechanisms of differentiation among farming systems, such as biophysical and socio-economic conditions. That hypothesis is based both on the typology objective and on prior expert knowledge and theories of the farm diversity in the study area. We present a methodological framework that aims to integrate participatory and statistical methods for hypothesis-based typology construction. This is an iterative process whereby the results of the statistical analysis are compared with the reality of the target population as hypothesized by the local experts. Using a well-defined hypothesis and the presented methodological framework, which consolidates the hypothesis through local expert knowledge for the creation of typologies, warrants development of less subjective and more contextualized quantitative farm typologies.

## Introduction

Smallholder farming systems are highly heterogeneous in many characteristics such as individual farming households’ land access, soil fertility, cropping, livestock assets, off-farm activities, labour and cash availability, socio-cultural traits, farm development trajectories and livelihood orientations, e.g. [1, 2]. Farm typologies can help to summarize this diversity among farming systems. Typology construction has been defined as a process of classification, description, comparison and interpretation or explanation of a set of elements on the basis of selected criteria, allowing reduction and simplification of a multiplicity of elements into a few basic/elementary types ([3] cited by [4]). As a result, farm typologies are a tool to comprehend the complexity of farming systems by providing a simplified representation of the diversity within the farming system by organizing farms into quite homogenous groups, the farm types. These identified farm types are defined as a specific combination of multiple features [5–7].

Capturing farming system heterogeneity through typologies is considered as a useful first step in the analysis of farm performance and rural livelihoods [8–9]. Farm typologies can be used for many purposes, for instance i) the selection of representative farms or prototype farms as case study objects, e.g. [10–12]; ii) the targeting or fine-tuning of interventions, for example by identifying opportunities and appropriate interventions per farm type, e.g. [13–18]; iii) for the extension of technologies, policies or ex-ante impact assessments to larger spatial or organizational scales (up-scaling and/or out-scaling), e.g. [19–22]; and iv) to support the identification of farm development trajectories and evolution patterns, e.g. [23–28].

Various approaches can be used to develop farm typologies [29]. The identification of criteria defining a farm type can be based on the knowledge of local stakeholders, such as extension workers and/or farmers, or derived from the analysis of data collected using farm household surveys which provide a large set of quantitative and qualitative variables to describe the farm household system [30]. Perrot et al. [26] proposed to define "aggregation poles" with local experts, i.e. virtual farms summarising the discriminating characteristics of a farm type, which can then be used as reference for the aggregation (manually or with statistical techniques) of actual farming households into specific farm types. Based on farm surveys and interviews, Capillon [6] used a (manual) step-by-step comparison of farm functioning to distinguish different types; this analysis focused on the tactical and strategic choices of farmers and on the overall objective of the household. Based on this approach, farm types were created using statistical techniques to first group farms according their structure, then within each of these structural groups, define individual farm types on the basis of their strategic choices and orientation [31]. Landais et al. [32] favoured the comparison of farming practices for the identification of farm types. Kostrowicki and Tyszkiewicz [33] proposed the identification of types based on the inherent farm characteristics in terms of social, organizational and technical, or economic criteria, and then representing these multiple dimensions in a typogram, i.e. a multi-axis graphic divided into quadrants, similar to a radar chart. Nowadays, statistical techniques have largely replaced the manual analysis of the survey data and the manual farm aggregation/comparison. Statistical techniques using multivariate analysis are one of the most commonly applied approaches to construct farm typologies, e.g. [34–41]. These approaches apply data-reduction techniques, i.e. combining multiple variables into a smaller number of ‘factors’ or ‘principal components’, and clustering algorithms on large databases.

Typologies are generally conditioned by their objective, the nature of the available data, and the farm sample [42]. Thus, the methodological decisions on data collection, variable selection, data-reduction and clustering have a large impact on the resulting typology. Furthermore, typologies tend to remain a research tool that is not often used by local stakeholders [42]. In order to make typologies more meaningful and used, we argue that typology development should involve local stakeholders (iteratively) and be guided by a hypothesis on the local agricultural features and the criteria for differentiating farm household systems. This hypothesis can be based on perceptions of, and theories on farm household functioning, constraints and opportunities within the local context, and the drivers and mechanisms of differentiation [43–44]. Drivers of differentiation can include biophysical conditions, and the variation therein, as well as socio-economic and institutional conditions such as policies, markets and farm household integration in value chains.

The objective of this article is to present a methodological approach for typology construction on the basis of an explicit hypothesis. Building on a case study of Zambia, we investigate how typology users’—here, two development projects—objectives and initial hypothesis regarding farm household diversity, impacts typology construction and consequently, its results. Based on this we propose a methodological framework for typology construction that utilizes a combination of expert knowledge, participatory approaches and multivariate statistical methods. We further discuss how an iterative process of hypothesis-refinement and typology development can inform participatory learning and dissemination processes, thus fostering specific adoption in addition to the fine-tuning and effective out-scaling of innovations.

## Materials and methods

### Typology construction in the Eastern Province, Zambia

We use a sample of smallholder farms in the Eastern Province of Zambia to illustrate the importance of hypothesis formulation in the first stages of the typology development. This will be done by showing the effects of using different hypotheses on the typology construction process and its results, while using the same dataset. Our experience with typology construction with stakeholders in Zambia made clear that i) the initial typology objective and hypotheses were not clearly defined nor made explicit at the beginning of the typology development, and ii) iterative feedbacks with local experts are needed to confirm the validity of the typology results.

**Fig 1:**
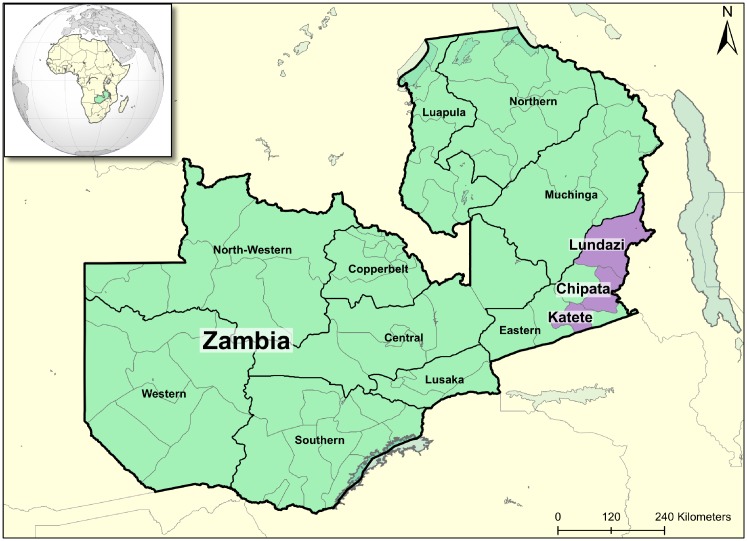
Map of the study area: Lundazi, Chipata and Katete districts (in violet), Eastern Province of Zambia.

The typology construction work in the Eastern Province of Zambia (Fig 1) was performed for a collaboration between SIMLEZA (Sustainable Intensification of Maize-Legume Systems for the Eastern Province of Zambia) and Africa RISING (Africa Research in Sustainable Intensification for the Next Generation; https://africa-rising.net/); two research for development projects operating in the area. Africa RISING is led by IITA (International Institute of Tropical Agriculture; http://www.iita.org/) and aims to create opportunities for smallholder farm households to move out of hunger and poverty through sustainably intensified farming systems that improve food, nutrition, and income security, particularly for women and children, and conserve or enhance the natural resource base. SIMLEZA is a research project led by CIMMYT (International Maize and Wheat Improvement Center; http://www.cimmyt.org/) which, amongst other objectives, seeks to facilitate the adoption and adaptation of productive, resilient and sustainable agronomic practices for maize-legume cropping systems in Zambia’s Eastern Province. The baseline survey data that was used was collected by the SIMLEZA project in 2010/2011. The survey dataset (S1 Dataset) was used to develop three typologies using three different objectives, to investigate the effects that different hypotheses have on typology results.

Zambia’s Eastern Province is located on a plateau with flat to gently rolling landscapes at altitudes between 900 to 1200 m above sea level. The growing season lasts from November to April, with most of the annual rainfall of about 1000 mm falling between December and March [45]. Known for its high crop production potential, Eastern Zambia is considered the country’s ‘maize basket’ [46]. However, despite its high agricultural potential (Table 1), the Eastern Province is one of the poorest regions of Zambia, with the majority of its population living below the US$1.25/day poverty line [47].

**Table 1:** Main farming characteristics of three districts of Eastern Province of Zambia, Lundazi, Chipata and Katete.

Characteristics	Unit	Lundazi	Chipata	Katete
***Climate***	-	Tropical Savanna	Tropical Savanna	Humid Subtropical
***Precipitation***^1^	*mm/year*	896	1 023	1 090
***Average temperature***^2^	*°C*	19.1–27.0	18.0–25.3	17.4–25.6
***Altitude***	*masl*	1 143	1 140	1 060
***Population density***^3^	*persons/km*^*2*^	22.4	67.6	60.4
***Main Food crops***^4^	*from most to least frequent*	Maize Groundnut Beans	Maize Groundnut Beans	Maize Groundnut Cowpea
***Main Cash crops***^4^	*from most to least frequent*	Cotton Sunflower Tobacco	Sunflower Cotton Tobacco	Cotton Sunflower Tobacco
***Livestock kept***^4^	*from most to least frequent*	Chickens Cattle Pigs Goats	Chickens Pigs Goats Cattle	Chickens Pigs Cattle Goats

^1^: Average precipitation (cumulated annual rainfall) from weather data was collected between 1982 and 2012. Source: http://en.climate-data.org/region/1612/;

^2^: Lowest monthly average temperature and warmest monthly average temperature. Source: http://en.climate-data.org/region/1612/;

^3^: Source: http://www.zamstats.gov.zm/;

^4^: Sources: SIMLEZA Baseline Survey 2011–2012.

The SIMLEZA baseline survey captured household data of about 800 households in three districts, Lundazi, Chipata and Katete (Fig 1). Although smallholder farmers in these districts grow similar crops, including maize, cotton, tobacco, and legumes (such as cowpeas and soy beans), the relative importance of these crops, the livestock herd size and composition, and their market-orientation differ substantially, both between and within districts. The densely populated Chipata and Katete districts (respectively, 67.6 and 60.4 persons/km^2^) [48] located along the main road connecting the Malawian and Zambian capital cities are characterised by highly intensive land use, relatively small land holdings and relatively small livestock numbers. The Lundazi district, by contrast, has rather extensive land-use and a low population density (22.4 persons/km^2^) [48], and is characterised by large patches of unused and fallow lands, which are reminiscent of land-extensive slash and burn agriculture.

### Alternative typology objectives and hypotheses

Iterative consultations with some of the SIMLEZA-project members in Zambia, informed the subsequent construction of three farm household typologies, all based on different objectives. The objective of the first typology (T1) was to classify the surveyed smallholder farms on the basis of the most distinguishing features of the farm structure (including crop and livestock components). The first hypothesis was that farm households could be grouped by farm structure, captured predominantly in terms of wealth indicators such as farm and herd size. When the resulting typology was not deemed useful by the local project members (because it did not focus enough on the cropping activities targeted by the project), a second typology was constructed with a new objective and hypothesis. The objective of the second typology (T2) was to differentiate farm households in terms of their farming resources (land and labour) and their integration of grain legumes (GL). The second hypothesis was that farming systems could be grouped according to their land and labour resources and their use of legumes, highlighting the labour and land resources (or constraints) of the groups integrating the most legumes. But again the resulting typology did not satisfy the local project members; they expected to see clear differences in the typology results across the three districts (Lundazi, Chipata and Katete), as the districts represented rather different farming contexts. Thus for the third typology (T3), the local partners hypothesized that the farm types and the possibilities for more GL integration would be strongly divergent for the three districts, due to differences in biophysical and socio-economic conditions (Table 1). The hypothesis used was that the farm households could be grouped according to their land and labour resources and their use of legumes and that the resulting types would differ between the three districts. Therefore, the objective of the third typology focused on GL integration as for T2, but for the three districts separately (T3-Lundazi, T3-Chipata and T3-Katete).

### Multivariate analysis on different datasets

On the basis of the household survey dataset, five sub-databases were extracted which corresponded to the three subsets of variables chosen to address the different typology objectives (Table 2). The first two sub-databases included all three districts (T1 and T2) and the last three sub-databases corresponded to the subdivision of the data per district (T3). In each sub-database, some surveyed farms were identified as outliers and others had missing values; these farms were excluded from the multivariate analysis. A Principal Component Analysis (PCA) was conducted to reduce each dataset into a few synthetic variables, i.e. the first principal components (PCs). This was followed by an Agglomerative Hierarchical Clustering using the Ward’s minimum-variance method, which was applied on the outcomes of the PCA (PCs’ scores) to identify clusters. The Ward’s method minimizes within-cluster variation by comparing two clusters using the sum of squares between the two clusters, summed over all variables [49]. The number of clusters (i.e. farm types) was defined using the dendrogram shape, in particular the decrease of the dissimilarity index (“Height”) according to the increase of the number of clusters. The resulting types were interpreted by the means of the PCA results and put into perspective with the knowledge of the local reality. All the statistical analyses were executed in R (version 3.1.0, *ade4* package; [50]).

**Table 2:** Surveyed variables from the Eastern Province of Zambia and for the three districts (Lundazi, Chipata and Katete) and the variables used for the three Eastern Zambia typologies (T1, T2 and T3).

Variables				T1	T2	T3	Eastern Province	Lundazi	Chipata	Katete
				Crop-livestock structure	Farming resources and legume use					
Category	Code	Description	Unit				Mean (min-max)	Mean	Mean	Mean
***Structure***	*hhsize*	*Number of member in the household*	*number*	**x**			**6.9** (1–20)	**7.4**	**6.9**	**6.3**
	*oparea*	*Total operated area by the farm*	*ha*	**x**	**x**	**x**	**4.8** (0.02–35)	**6.1**	**3.9**	**4.6**
	*tlu*	*Total tropical livestock unit*	*tlu*	**x**	**x**	**x**	**3.1** (0–29)	**3.5**	**2.3**	**4.1**
***Livestock***	*cattle*	*Number of cattle*	*tlu*				**2.1** (0–24)	**2.8**	**1.3**	**2.6**
	*cattleratio*	*Share of cattle in the total herd*	-	**x**			**0.36** (0–1)	**0.43**	**0.27**	**0.43**
	*smallrum*	*Number of small ruminants (goats& sheeps)*	*tlu*				**0.2** (0–5)	**0.2**	**0.2**	**0.2**
	*smallrumratio*	*Share of small ruminants in the total herd*	-	**x**			**0.10** (0–1)	**0.07**	**0.17**	**0.04**
	*pig*	*Number of pigs*	*tlu*				**0.6** (0–8)	**0.5**	**0.6**	**1.1**
	*pigratio*	*Share of pigs in the total herd*	-	**x**			**0.23** (0–1)	**0.15**	**0.24**	**0.34**
	*chicken*	*Number of chickens*	*tlu*				**0.1** (0–1.8)	**0.1**	**0.1**	**0.1**
	*chickenratio*	*Share of poultry in the total herd*	-	**x**			**0.25** (0–1)	**0.30**	**0.28**	**0.12**
***Labour***	*totlabour*	*Total labour use in the farm per year*	*hours*	**x**	**x**	**x**	**613** (7–5 531)	**642**	**624**	**546**
	*hiredcost*	*Total hired cost per year*	*kZKW*	**x**	**x**	**x**	**236** (0–2 470)	**354**	**169**	**173**
	*femratio*	*Share of the total labour done by women*	-			**x**	**0.52** (0–1)	**0.52**	**0.51**	**0.53**
	*preplabrat*	*Share of the total labour allocated for land preparation*	-		**x**	**x**	**0.16** (0–1)	**0.17**	**0.18**	**0.13**
	*weedlabrat*	*Share of the total labour allocated for weeding*	-		**x**	**x**	**0.33** (0–0.8)	**0.31**	**0.33**	**0.38**
	*harvlabrat*	*Share of the total labour allocated for harvesting*	-				**0.33** (0–0.9)	**0.30**	**0.34**	**0.36**
	*shelabrat*	*Share of the total labour allocated for threshing and shelling*	-				**0.17** (0–0.8)	**0.22**	**0.15**	**0.13**
***Income***	*totincome*	*Total income per year*	*kZKW*			**x**	**10 522** (0–112 751)	**14 292**	**7 400**	**9 701**
	*cropincome*	*Income generated per year from cropping activities*	*kZKW*	**x**	**x**		**6 749** (0–94 852)	**9 553**	**4205**	**7063**
	*offincome*	*Income generated per year from off-farm activities*	*kZKW*	**x**	**x**		**3 256** (0–96 000)	**4 456**	**2770**	**2215**
	*anlincome*	*Income generated per year from livestock activities*	*kZKW*				**517** (0–31852)	**682**	**425**	**424**
	*cropincratio*	*Share of the total income generated per year by cropping activities*	-			**x**	**0.69** (0–1)	**0.71**	**0.64**	**0.74**
	*anlincratio*	*Share of the total income generated per year by livestock activities*	-			**x**	**0.08** (0–1)	**0.06**	**0.09**	**0.07**
	*offincratio*	*Share of the total income generated per year by off-farm activities*					**0.24** (0–1)	**0.23**	**0.27**	**0.19**
***Legume***	*legratio*	*Percentage of the total operated area allocated for leguminous crops*	*%*		**x**	**x**	**20.1** (0–100)	**22.0**	**21.2**	**14.9**
	*legexp*	*Number of years of experience on leguminous cropping*	*year*		**x**		**6.4** (0–73)	**6.1**	**7.0**	**5.9**
	*legscore*	*Farmer evaluation of his/her leguminous cropping activities as a measure of their satisfaction*	-		**x**		**4.0** (0–5)	**4.0**	**4.0**	**3.9**

kZKW = 1000 x ZKW (Zambian Kwacha); 1 USD ≈ 13 100 ZKW

## Results and discussion on the contrasting typologies

**Fig 2:**
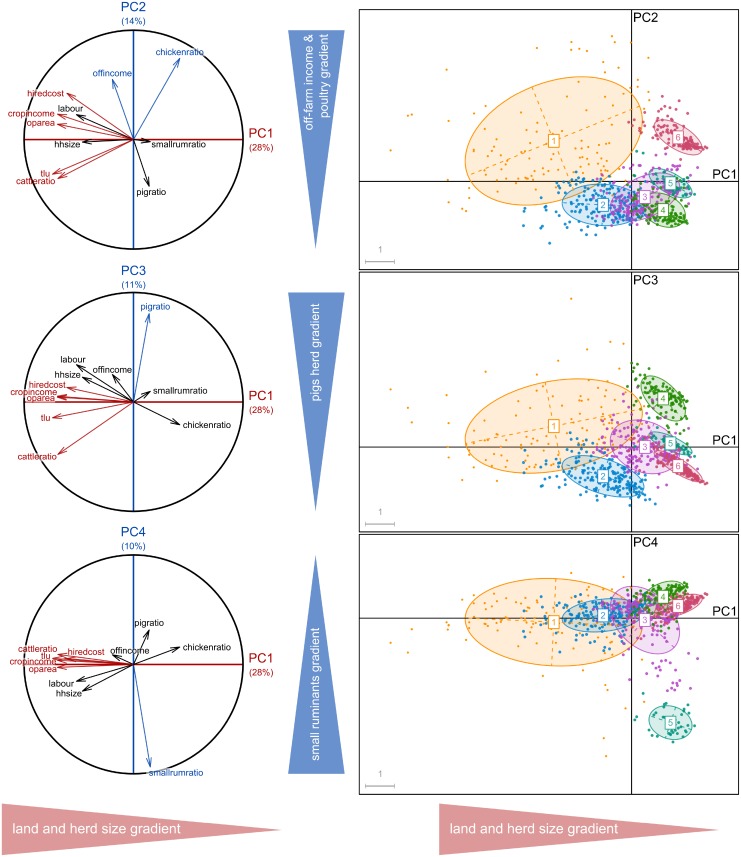
Typology 1: Representation of the six farm types of resulting from the Principal Component Analysis and clustering analysis on the planes defined by the first four principal components. The red colour variables are the most explanatory of the horizontal axis (PC1); those in blue are the most explanatory variables of vertical axes (PC2, PC3 and PC4), thus defining the gradients.

**Fig 3:**
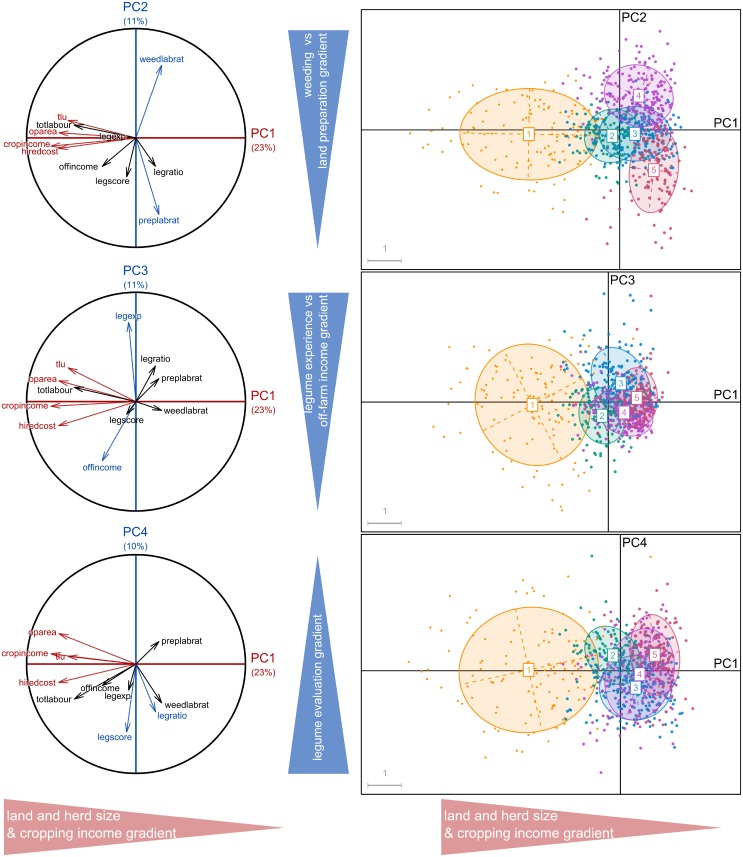
Typology 2: Representation of the five farm types of resulting from the Principal Component Analysis and clustering analysis on the planes defined by the first four principal components. The red colour variables are the most explanatory of the horizontal axis (PC1); those in blue are the most explanatory variables of vertical axes (PC2, PC3 and PC4), thus defining the gradients.

**Fig 4:**
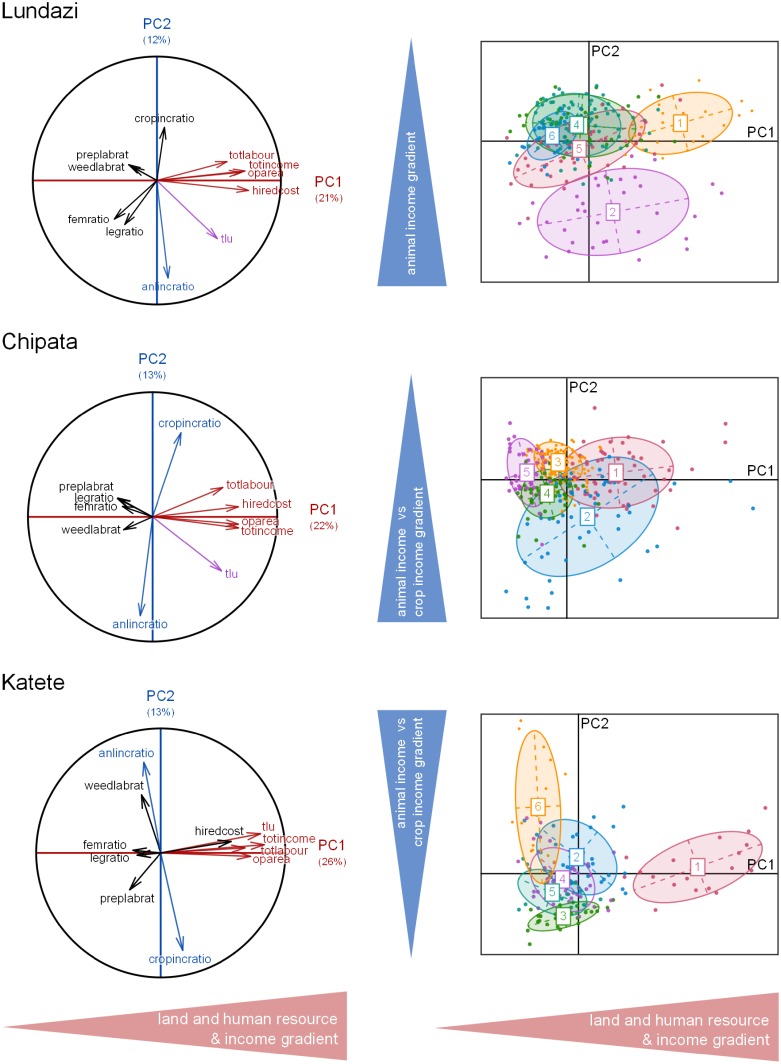
Typology 3: Representation of the farm types of resulting from the Principal Component Analysis and clustering analysis on the planes defined by the first four principal components, for the districts Lundazi, Chipata and Katete. The red coloured variables are the most explanatory of the horizontal axis (PC1); those in blue are the most explanatory variables of vertical axes (PC2) and those in violet are variables correlated with both PC1 and PC2.

Of the five PCAs, the first four principal components explained between 55% and 64% of the variability in the five sub-databases (64, 55, 55, 57 and 62% for respectively T1, T2, and T3-Lundazi, T3-Chipata and T3-Katete). The four PCs are most strongly correlated to variables related to farm structure, labour use and income. The variables most correlated with PC1 were the size of the farmed land (*oparea*; five PCAs), the number of tropical livestock units (*tlu*; four PCAs), the cost of the hired labour (*hirecost*; four PCAs) and total income or income generated by cropping activities (*totincome* or *cropincome*; five PCAs) (Figs 2, 3 and 4).

The following discriminant dimensions were more related to the specific objective of each typology. For the typology T1, PC1, PC2, PC3 and PC4 were related to the most important livestock activity (i.e. contribution of each livestock type to the total tropical livestock units (TLU) represented by *cattleratio*, *chickenratio*, *pigratio* and *smallrumratio* respectively), thus distinguishing the farms by their dominant livestock type (Fig 2). The six resulting farm types are organized along a land and TLU gradient, from type 1 (larger farms) to type 6 (smaller farms). In addition to land and TLU, the farm types differed according their herd composition: large cattle herds for type 1 and type 2, mixed herds of cattle and small ruminants or pig for type 3, mostly pigs for type 4, small ruminant herds for type 5 and finally, mostly poultry for type 6 (Fig 2).

For the typology T2, the labour constraints for land preparation (*preplabrat*) and weeding (*weedlabrat*) determined the second discriminant dimension (PC2), while the legume features (experience, legume evaluation and cropped legume proportion represented by *legexp*, *legscore* and *legratio* respectively) only appeared correlated to PC3 or PC4. However, these two last dimensions were not useful to discriminate the surveyed farms, since the farm types tended to overlap in PC3 and PC4 (Fig 3). Therefore, while these were variables of interest (i.e. targeted in the T2-typology objective), no clear difference or trend across farm types was identified for the legume features in the multivariate results (Fig 3). The five resulting farm types were also organized along a land and TLU gradient, which was correlated with the income generated per year from cropping activities (*cropincome*) and the hired labour (*hiredcost*), ranging from type 1 (higher resource-endowed farms employing a large amount of external labour) to type 5 (resource-constrained farms, using almost only family labour). Furthermore, type 4 and type 5 were characterized by their most time-consuming cropping activity, weeding and soil preparation respectively (Fig 3).

For the typology T3, Lundazi, Chipata and Katete farms tended to primarily be distinguished according to a farm size, labour and income gradients (Fig 4). The number of the livestock units (*tlu*) remained an important discriminant dimension that was correlated to either PC1 or PC2 in the three districts (Fig 4). Although the selection of the variables was made to differentiate the farmers according to their legume practices (*legratio*), this dimension appeared only in PC3 or PC5, explaining less than 12% of the variability surveyed. Moreover, similarly to T2, the farm types identified were not clearly distinguishable on these dimensions. Thus, besides the clear differences among farms in terms of their land size, labour and income (PC1), farms were primarily segregated by their source of income, i.e. cropping activities (*cropincratio*) vs. animal activities (*anlincratio*) (Fig 4). In T3-Lundazi, T3-Chipata and T3-Katete, the resulting farm types were also organized along a resource-endowment gradient, from type 1 (higher resource-endowed farms) to type 6 (resource-constrained farms). Additionally, they were distinguished by their main source of income: i) for T3-Lundazi, large livestock sales for type 2, mostly crop products sales (low livestock sales) for types 1, 3, 4, and 6, and off-farm activities for type 5; ii) for T3-Chipata, crop revenues for type 3, livestock sales for type 2 and mixed revenues from crop sales and off-farm activities for type 1, 4 and 5; iii) for T3-Katete, crop revenues for types 3 and 5, mixed revenues from crop sales and off-farm activities for type 1, 2 and 4, and mixed revenues from livestock sales and off-farm activities for type 6 (Fig 4).

**Fig 5:**
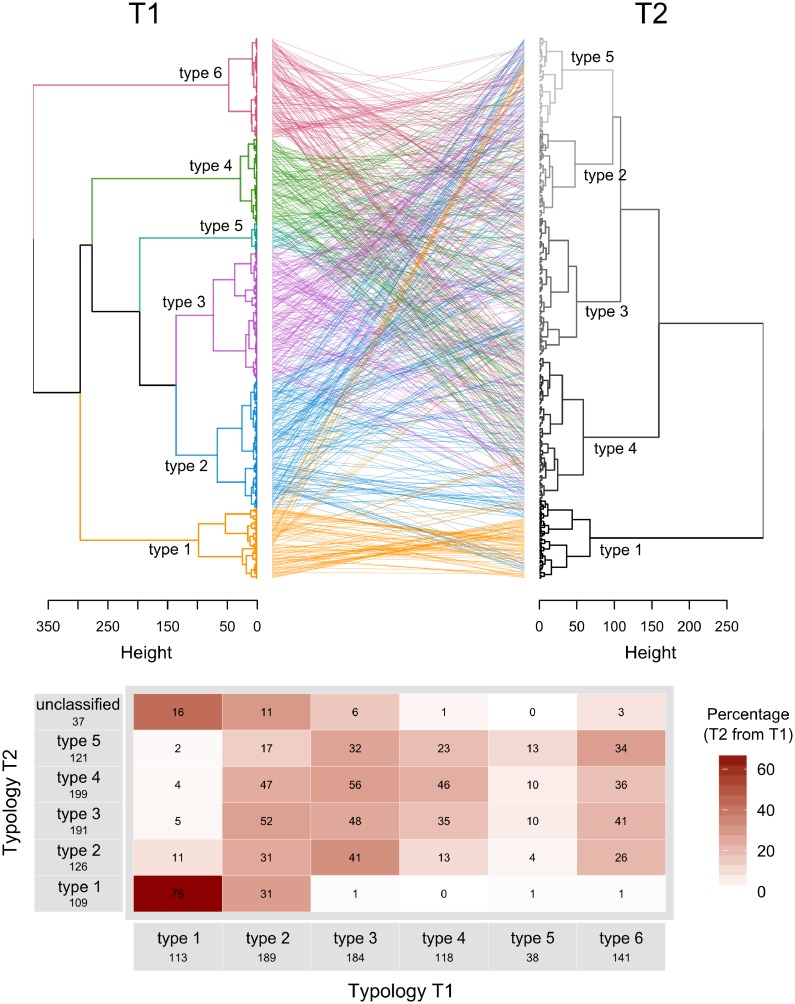
(a) Comparison of the two dendrograms from the resource-based typology (T1) and the crop-based typology (T2), and (b) cross-tabulation of numbers of farms of T1 allocated to different types of T2; the intensity of the red colouring indicates the percentage of overlap. The ‘unclassified’ farms are farms that were included in T1 but were detected as outliers for T2. Fig 6a illustrates the overlapping between T1 and T2, comparing the individual position each farm in the two dendrogram of the two typologies, while Fig 6b quantifies the percentage of overlap between the two typologies.

**Fig 6:**
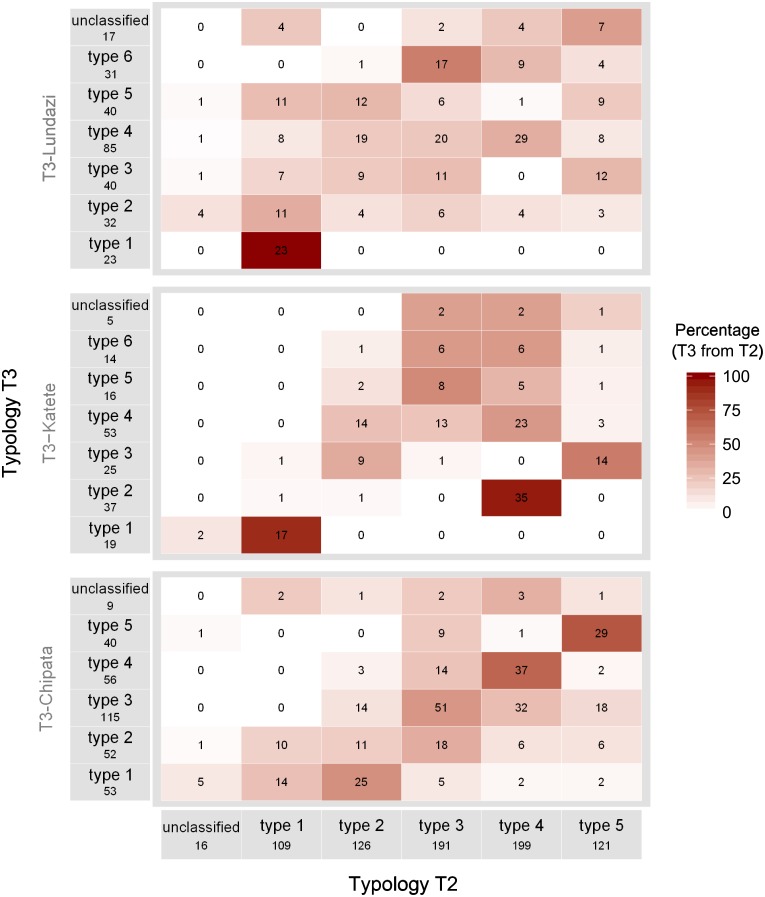
Cross-tabulations of numbers of farms of typology T2 allocated to different types of typologies for districts Lundazi (T3-Lundazi; a), Chipata (T3- Chipata; b) and Katete district (T3- Katete; c). The intensity of the red colouring indicates the percentage of overlap.

The overlap of the typologies is presented in Figs 5 and 6. A strong overlap is indicated by a high percentage (and darker shading) in only one cell per row and column (Figs 5b and 6). The overlap between the presented typologies was not clear (Figs 5 and 6) despite the importance of farm size, labour and income in the first principle component (PC1) in all typologies. The best overlap was observed between the typology T2 and the typology T3 for the Chipata district (T3-Chipata). Moreover, the types 1 (i.e. farms with larger farm area, higher income and more labour used) overlapped between typologies: 69% of type 1 from T2 belonged to type 1 from T1 (Fig 5) and, 100 and 89% of the types 1 from Lundazi and Katete, respectively, belonged to type 1 from T2 (Fig 6). The majority of the unclassified farms (i.e. farms present in T1 but detected as outliers in T2 and T3) were related to the ‘wealthier’ types, type 1 and type 2 (Figs 5 and 6).

For the all the typologies (T1, T2, T3-Lundazi, T3-Chipata and T3-Katete), the main discriminating dimension was related to resource endowment: farm structure in terms of land area and/or animal numbers, labour use and income, which has been observed in many typology studies. In this case, the change in typology objective and the corresponding inclusion of variables from the dataset on legume integration (e.g. *legratio*) did not result in a clearer separation among farm types in T2 when compared to T1. The importance of farm structure variables in explaining the datasets’ variability (Figs 2, 3 and 4) resulted in overlap among typologies regarding the larger, more well-endowed farms, that comprised ca. 10% of the farms, but for types representing medium- and resource-constrained farms the overlap between typologies was limited (Figs 5 and 6).

**Fig 7:**
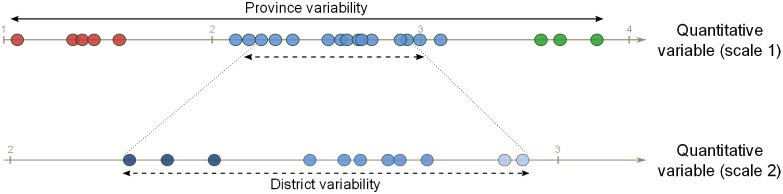
Theoretical example of a change of scale, from scale 1 to scale 2 (e.g. from province to district). Distribution of observations of a quantitative variable (e.g. farm area) at the province level (level 1) and at the district level (level 2). The different colours are associated with different values classes within the variable. Zooming in from scale 1 to scale 2, magnifies the variation within the district, potentially revealing new classes.

The difference between typologies T2 and T3 relates to a scale change, i.e. from province to district scale. Zooming in on a smaller scale allows amplification of the local diversity. Indeed, the range of variation could be different at provincial level (i.e. here three districts were merged) when compared to the district level (Table 1). Thus narrowing the study scale makes intra-district variability more visible, and potentially reveals new types leading to a segregation/splitting of one province-level type into several district-level types (Fig 7). The differences between typologies that arise from scale differences highlight the importance of scale definition when investigating out-scaling and up-scaling of target interventions.

## Methodological framework for typology construction

**Fig 8:**
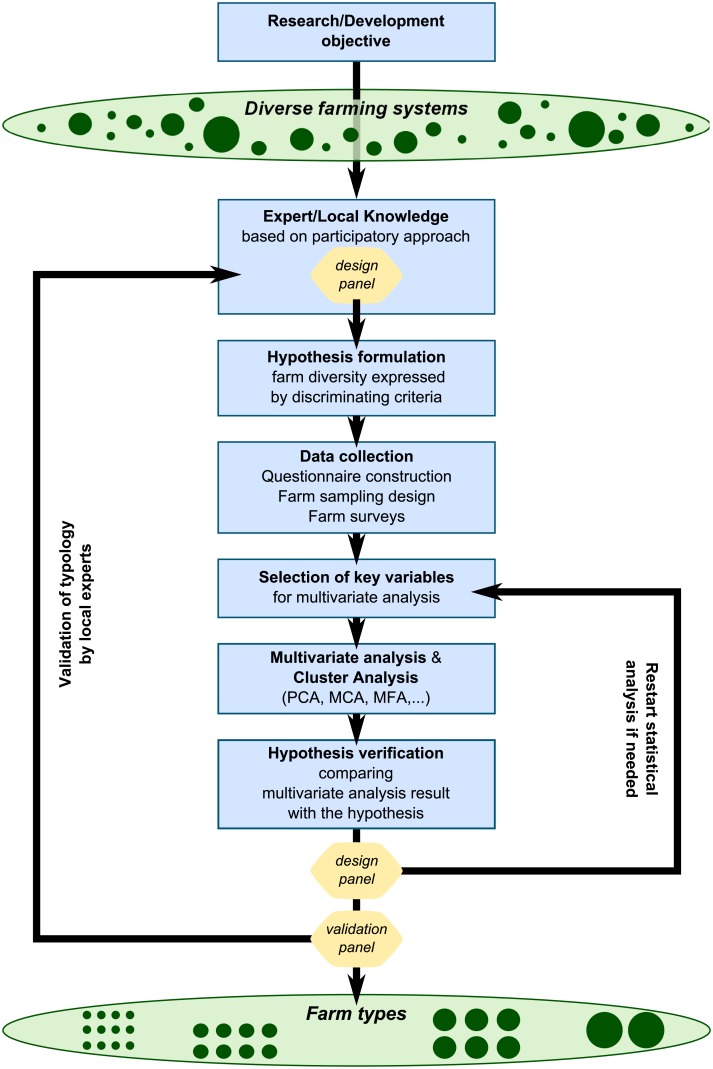
General framework of the typology process, where expert knowledge is combined with statistical techniques (PCA: Principal Component Analysis; MCA: Multiple Correspondence Analysis; MFA: Multiple Factorial Analysis).

The proposed methodological framework (Fig 8) aims to integrate statistical and participatory methods for hypothesis-based typology construction using quantitative data, to create a typology that is not only statistically sound and reproducible but is also firmly embedded in the local socio-cultural, economic and biophysical context. From a heterogeneous population of farms to the grouping into coherent farm types, the step-wise structure of this typology construction framework comprises the following steps: i) precisely state the objective of the typology; ii) formulate a hypothesis on farming systems diversity; iii) design a sampling method for data collection; iv) select the variables characterizing the farm households; v) cluster the farm households using multivariate statistics; and vi) verify and validate the typology result with the hypothesis and discuss the usability of the typology with (potential) typology users. This step-wise process can be repeated if the multivariate analysis results do not match the diversity of the targeted population as perceived by the validation panel and typology users (Fig 8).

### Typology objectives, target population and expert panel

A farm typology is dependent on the project goals and the related research, innovation or development question [39], which determine the typology objective. This will affect the delineation of the system under study, i.e. the target population size, in socio-institutional and geographical dimensions. The socio-institutional aspects that affect the size of the target population include criteria such as the type of entities involved (e.g., farms, rural households or individual farmers) and some initial cut-off criteria. These cut-off criteria can help in reducing the population size, such as a minimum or maximum structural size or the production orientation (e.g., food production, commercial and/or export-oriented; conventional or organic). The geographical dimension will affect the size of the target population by determining the spatial scale of the study, which in turn can be influenced by natural or administrative boundaries or by biophysical conditions such as suitability for farming. The scale at which the study is conducted can amplify or reduce the diversity that is encountered (Fig 7).

Stakeholders (including farmers) with a good knowledge of the local conditions and the target population and its dynamics can inform the various steps of the typology development, forming an expert panel for consultation throughout the typology construction process. The composition of the panel can be related to the objective of the typology. Existing stakeholder selection techniques, e.g. [51–52] can be used for the identification and selection of panel experts. The group of experts can be split into a ‘design panel’ that is involved in the construction of the typology, and a ‘validation panel’ for independent validation of the result (cf. Section ‘Hypothesis verification and typology validation’). Finally, involving local stakeholders who are embedded in the target population may trigger a broader local involvement in the research process, facilitating data collection and generating more feedback and acceptance and usability of the results [43].

### Hypothesis on typology structure

A multiplicity of typologies could describe the same faming environment depending on the typology objective and thus the selected criteria for typology development [43]. In the proposed framework (Fig 8), the typology development is based on the formulation of a hypothesis on the diversity of the target population by the local experts, the design panel, in order to guide the selection of variables to be used in the multivariate statistical analysis. The hypothesis relates to the main features of local agriculture, stakeholder assumptions and theories on farm functioning and livelihood strategies in the local context, and on their interpretation of the relevant external forces and mechanisms that can differentiate farm households. Heterogeneity can emerge in response to very diverse socio-cultural, economic and biophysical drivers that can vary in significance within the studied region. In addition to the primary discriminatory features, the hypothesis can also make the following features explicit; the most prominent types of farms that are expected, their relative proportions, the most crucial differences between the farm types, the gradients along which the farms may be organized and possible relationships or correlations between specific farm characteristics. These perceptions and theories about the local diversity in rural livelihoods and farm enterprises are often present but are not always made explicit; the hypothesis formulation by the design panel is meant to make these explicit and intelligible to the external researchers. Hence, the design panel is expected to reflect on the drivers and features of the farm diversity encountered in the targeted population and reach a consensus on the main differentiating criteria and, ideally, have a preliminary inventory of the expected farm types.

An example of a hypothesis formulated by local experts could be that farms are distinguished by the size of the livestock herd, their reliance on external feeds and their proximity to livestock sale-yards; thus, there may be a gradient from large livestock herds, very reliant on external feeds, and close the sale-yards, to small herds, less reliant on external feeds further away from sale-yards. The discussions of the design panel are guided by the general typology objective. The hypothesis can further be informed by other participatory methods, previous studies in the area or by field observations. This allows for a wide range of information to be used for the hypothesis consolidation. Most of the information compiled in the formulated hypothesis is qualitative, but can also be informed by maps and spatial data in geographical information systems. The statistical analysis that follows will use quantitative features and boundaries of the farm entities in the study region.

### Data collection, sampling and key variables selection

The creation of a database on the target population is an essential step in the typology construction based on quantitative methods. The farm sampling needs to capture the diversity of the target population [41]. The size of the sample and the sampling method [53] affect the proportion of farms belonging to each resulting farm type; for instance a very small farm type is likely to be absent in a reduced sample. Thus the sampling process, notably the choice of sample size, should be guided by the initial hypothesis.

The survey questionnaire needs to reflect the hypothesis formulated in the previous step, i.e. containing at least the main features and differentiation criteria listed by the design panel. However, the survey can be designed to capture the entire farming system [1, 8], collecting information related to all its components (i.e. household/family, cropping system, livestock system), their interactions, and the interactions with the biophysical environment in which the farming system is located (e.g. environmental context, economic context, socio-cultural context). The anticipated analytical methods to be applied, especially the multivariate techniques, also guide decisions about the nature of data (e.g. categorical or continuous data) to collect.

Finally, the selection of key variables for the multivariate analysis is adapted to the typology objective following the previous step of exchanges with the expert panel and hypothesis formulation. Together researchers and the expert design panel select the key variables that correspond to the formulated hypothesis. These selected key variables constitute a sub-database of the collected data, which will be used for the multivariate analysis. Kostrowicki [54] advised to favour integrative variables (i.e. combining several attributes) rather than elementary variables. The number of surveyed entities has to be larger than the number of key variables; a factor five is often advised [49].

### Multivariate statistics

Multivariate statistical analysis techniques are useful to identify explanatory variables (discriminating variables) and to group farms into homogeneous groups that represent farm types. A standard approach is to apply a data-reduction method on the selected set of variables (key variables) to derive a smaller set of non-correlated components or factors. Then clustering techniques are applied to the coordinates of the farms on these new axes. Candidate data-reduction techniques include: i) Principal Component Analysis for quantitative (continuous or discrete) variables, e.g. [1, 36, 55]; ii) Multiple Correspondence Analysis for categorical variables, e.g. [33]; iii) Multiple Factorial Analysis for categorical variables organized in multi-table and multi-block data sets, e.g. [34]; iv) Hill and Smith Analysis for mixed quantitative and qualitative variables, e.g. [27]; v) Multidimensional scaling to build a classification configuration in a specific dimension, e.g. [41, 56]; or vi) variable clustering to reduce qualitative and quantitative variables into a small set of (quantitative) “synthetic variables” used as input for the farm clustering, e.g. [57]. Although the number of key variables is reduced, the variability of the dataset is largely preserved. However, as a result of the multivariate analysis, not all the key variables selected will necessarily be retained as discriminating variables.

Subsequently, a classification method or clustering analysis (CA) can be applied on these components or factors to identify clusters that minimize variability within clusters and maximize differences between clusters. There are two methods of CA commonly used: i) Non-hierarchical clustering, i.e. a separation of observations/farms space into disjoint groups/types where the number of groups (k) is fixed; and ii) Hierarchical clustering, i.e. a stepwise aggregation of observations/farms space into disjoint groups/types (first each farm is a group all by itself, and then at each step, the two most similar groups are merged until only one group with all farms remains). The Agglomerative Hierarchical Clustering algorithm is often used in the typology construction process, e.g. [24, 34, 35, 41, 55]. The two clustering methods can be used together to combine the strengths of the two approaches, e.g. [15, 58, 59]. When used in combination, hierarchical clustering is used to estimate the number of clusters, while non-hierarchical clustering is used to calculate the cluster centres. Some statistical techniques exist to support the choice of the number of clusters and to test the robustness of the cluster results, such as clustergrams, slip-samples or bootstrapping techniques [49, 60, 61]. The “practical significance” of the cluster result has to be verified [49]. In practice, a limited number of farm types is often preferred, e.g. three to five for Giller et al. [8], and six to fifteen for Perrot and Landais [42].

### Hypothesis verification and typology validation

The resulting farm types have to be conceptually meaningful, representative of and easily identifiable within the target population [62]. The farm types resulting from the multivariate and cluster analysis are thus compared with the initial hypothesis (cf. Section ‘Hypothesis on typology structure’; Fig 8), by comparing the number of types defined, their characteristics and their relative proportions in the target population. The correlations among variables that have emerged from the multivariate analysis can also be checked with local experts. This has to be part of an iterative process where the results of the statistical analysis are compared with the reality of the target population in discussion with the expert panels (Fig 8). When involved in this process, local stakeholders can help in understanding the differences between the hypothesis and the results of the statistical analysis. In the case of results that deviate from the hypothesis, the multivariate and cluster analysis may need to be repeated using a different selection of variables, by examining outliers or the distributions of the selected variables. The discussion and feedback sessions with local stakeholders (‘design panel’ of experts) may need to be re-initiated until no new information emerges from the feedback sessions. Later, the driving effects of external conditions (such as biophysical and socio-economic features) on farming systems differentiation can be tested statistically analysing the relationships between the resulting farm types and external features variables.

Finally, when the design panel recognizes the farm types identified with the statistics analysis, an independent validation of the typology results and its usability by potential users is desired (Fig 8). Preferably, to allow an independent verification of the constructed typology, a ‘validation panel’ should be independent of the design panel that formulated the hypothesis. The resulting typology is presented to the validation panel whose members are asked to compare it with their own knowledge on the local farming systems diversity. The objective of this last step is to, in hindsight, demonstrate that the simplified representation reflected in the typology is a reasonable representation of the target population and that the typology satisfies the project goals. Some criteria were proposed to support the validation process of the typology by the validation panel ([3] cited by [4]): i) *Clarity*–farm types should be clearly defined and thus understandable by the local stakeholders (including the validation panel); ii) *Coherence*–examples of existing farms should be identifiable by the local experts for each farm type, and, any gradient highlighted during the hypothesis formulation should be recognizable in the typology results; iii) *Exhaustiveness*–most of the target population should be included in the resulting farm types; iv) *Economy*–the typology should include only the necessary number of farm types to represent most of the target population diversity; and, v) *Utility and acceptability*–the typology should be accepted and judged as useful by the stakeholders (especially by the validation panel), for instance by providing diagnostics on the target population like the production constraints per identified farm type.

Thus, eventually the typology construction has gone through two triangulation processes: expert triangulation (by design panel and validation panel) and methodological triangulation (using statistical analysis and participatory methods).

## General discussion

### Importance of the learning process

The hypothesis-based typology construction process constitutes a learning process for the stakeholders involved such as local experts, local policy makers and research for development (R4D) project leaders, and for the research team that develops the typology. For the local stakeholders, the process could lead to a more explicit articulation of the perceived (or theorised) diversity within the farming population and use of the constructed typology. The process involves an exchange of ideas and notions, and provides incentives to find consensus among different perspectives. Obviously, the resulting typology itself allows for reflection on the actual differences between farming households and on opportunities for farm development. By recognizing different farm types and the associated distributions of characteristics, typologies could also help farmers to identify development pathways through a comparison of their own farm household system with others (*Where am I*?), identifying successful tactics and strategies of other farm types (*What can I change*?) and their performances (*What improvement can I expect*?).

The research team not only gains a quantitative insight into the diversity and its distribution from the developed typology, but also obtains a detailed qualitative view on the target population, particularly if selected farms representing the identified farm types are studied in more detail. Indeed, the interactions with local experts and discussions about the interpretation of the typology could also provide insights into, for instance, socio-cultural dynamics and power relations within the farming population and local institutions, as well as other aspects not necessarily collected during the survey. For example, social mechanisms can become more visible to the researcher when the relationships between farm types are described during the discussions with the expert panels.

### Farm/household dynamics

Farms are moving targets [8], while typologies based on one-time measurements or data collection surveys provide only a snapshot of farm situations at a certain period of time [54]. Due to farm dynamics, these typologies could become obsolete and hence it is preferable to regularly update typologies [28, 29].

However, it has been argued that typologies based on participatory approaches tend to be more stable in time [29], because they are more qualitative and therefore could also integrate the local background and accumulated experience from the local participants. Consequently, the resulting qualitative types change less over time, although individual farms may change from one farm type to another [26, 34]. Thus, the framework presented here would allow combining the longer-term (and more qualitative) vision of the local diversity from the local stakeholders including the general observed trend into the hypothesis formulation, and the shorter-term situation of individual households.

### Typologies as social constructs

It is important to recognize that typology construction is a social process, and therefore that typologies are social constructs. The perspectives and biases of the various stakeholders in the typology construction process, including methodological decision-making by the research team (such as the selection of the key variables, selection of principal components and clusters, and their interpretation, etc.) shape the resulting typologies, and subsequently their usability in research and policy making. Consequently, participatory typology construction may be considered as an outcome of negotiation processes between different stakeholders aiming to reach consensus on the interpretation of heterogeneity within the smallholder farming population [63]. The consensus-oriented hypothesis formulation described here is also a way to mitigate the dominance of particular stakeholders in shaping the typology constructing process. Multiple consultations, feedbacks to the local stakeholders and the typology validation by the independent assessors (the validation panel) further limit the dominant influence of more powerful stakeholders.

### Typology versus simpler farm classification

Taking into account multiple features of the farm household systems, typologies facilitate the comparison of these complex systems within a multi–dimensional space [7]. However, with multivariate analysis, the underlying structure of the data defines the ranking of dimensions in terms of their power to explain variability. Therefore, as shown previously (cf. Section ‘Results and discussion on the contrasting typologies’), there is no guarantee that the multivariate analysis will highlight one specific dimension targeted by the researcher or the intervention project. Thus, if the goal is simply to classify farms based on one or two dimensions, a simpler classification based only on one or two variables may suffice to define useful farm classes for the intervention project. For example, an intervention project focused on supporting new legume growers, could classify farm(er)s on their legume cultivated area and their years of experience with legume cultivation only. In that case, we would not use the term farm typology but rather farm classification.

### Farm types and individual farmers

Farm typologies are groupings based on some selected criteria and the farm types tend to be homogeneous in these criteria, with some intra-group variability. Thus, typologies are useful for gathering farmers for discussion such that one would have groups of farmers who manage their farms similarly, have similar general strategies, or face similar constraints and have comparable opportunities. This is how typologies can be especially helpful in targeting interventions to specific farm types. However, individual farm differences remain; criteria that were not included in the typology and also individual farmer characteristics, such as values, culture, background or personal goals and projects can account for the observed individual farm differences. Thus, when interacting with individual farmers, much more farm-specific, social (household and community) and personal features can arise, for example their risk aversion or other hidden (non-surveyed) issues that would influence their adoption of novel interventions. This highlights the intra-type heterogeneity and also exposes the potential pitfalls when targeting interventions to be adopted by farmers.

## Conclusion

Agricultural research and development projects that evaluate or promote specific agricultural practices and technologies usually provide a particular set of interventions, for instance oriented towards soil conservation, improvement of cropping systems or animal husbandry. The focus and aims of such projects shape also the differentiation of the project’s target population into farm types that are often used for targeting interventions. In addition, a project’s specific impact and out-scaling objectives influence the number of farmers targeted and the spatial scale at which the interventions need to be disseminated, thus influencing the farmer selection strategy. Constructing farm typologies can help to get a better handle on the existing heterogeneity within a targeted farming population. However, the methodological decisions on data collection, variable selection, data-reduction and clustering can bear a large impact on the typology construction process and its results. We argue that the typology construction should therefore be guided by a hypothesis on the diversity and distribution of the targeted population based both on the demands of the project and on prior knowledge of the study area. This will affect the farming household selection strategy, the data that will be collected and the statistical methods applied.

We combined hypothesis-based research, context specificities and methodological issues into a new framework for typology construction. This framework incorporates different triangulation processes to enhance the quality of typology results. First, a methodological triangulation process supports the fusion of i) ‘snapshot’ information from household surveys with ii) long-term qualitative knowledge derived from the accumulated experience of experts. This fusion results in the construction of a contextualized quantitative typology, which provides ample opportunities for exchange of knowledge between experts (including farmers) and researchers. Second, an expert triangulation process involving the ‘design panel’ and the ‘validation panel’, results in the reduced influence of individual subjectivity. As shown in the Zambian illustration, the typology results were highly sensitive to the typology objective and the corresponding selection of key variables, and scale of the study. Changing from one set of variables to another or, from one scale to another, resulted in the surveyed farms shifting between types (Figs 5 and 6). We have thus highlighted the importance of having a well-defined (and imbedded in local knowledge) typology objective and hypothesis at the beginning of the process. Taking into account both triangulation processes in the presented framework, we conclude that the framework facilitates a solid typology construction that provides a good basis for further evaluation of entry points for system innovation, exploration of tradeoffs and synergies between multiple (farmer) objectives and to inform decisions on improvements in farm performance.

## Supporting information

S1 Dataset Data used for the typology construction. (XLSX)
